# Assessing the Functional Status of Older Cancer Patients in an Ambulatory Care Visit

**DOI:** 10.3390/healthcare3030846

**Published:** 2015-09-18

**Authors:** Janine Overcash

**Affiliations:** College of Nursing, the Ohio State University, 1585 Neil Avenue, Columbus, OH 43220, USA; E-Mail: Overcash.1@osu.edu

**Keywords:** comprehensive geriatric assessment, functional status, geriatrics

## Abstract

Functional status assessment is a useful and essential component of the complete history and physical exam of the older patient diagnosed with cancer. Functional status is the ability to conduct activities that are necessary for independence and more executive activities, such as money management, cooking, and transportation. Assessment of functional status creates a portal into interpreting the health of in older persons. Understanding limitations and physical abilities can help in developing cancer treatment strategies, patient/family teaching needs, and in-home services that enhance patient/family care. This article will review the benefits of functional assessment, instruments that can be used during an ambulatory care visit, and interventions that can address potential limitations.

## 1. Introduction

Functional status scores are predictive of falls [1,2], longer hospital stays [3], disease-related symptoms [4], poor tolerance to chemotherapy [5,6], emotional concerns [7], mortality [8], and general health status [9]. People aged 80 years and over are more likely to exhibit poor functional status even in the absence of multiple diagnostic conditions or cognitive disability [10]. Functional impairment at age 70 predicts mortality and institutionalization [11] and is more helpful than chronological age when predicting treatment outcomes [12]. This article will review the benefits of functional assessment, instruments that can be used during an ambulatory care visit and interventions that can address potential limitations.

## 2. Descriptions of Functional Status

Many providers fail to perform a functional assessment on older patients in clinical practice, despite 42% of community-dwelling seniors [13] and 63% of hospitalized elders having functional deficits [14]. In 1991, a classic study found that 66% of providers failed to address functional limitations even when specifically reported by the patient during an office visit [15]. Currently, functional status assessments are routinely performed in areas such as emergency rooms [16], oncology clinics [17], hemodialysis centers [9] hospitals [18,19], and primary care clinics [20]. Clinical guidelines from the Hartford Institute for Geriatric Nursing [21] and the American Geriatrics Society [22] provide recommendations for assessment and management of functional limitations.

Traditional elements of functional status are bathing, dressing, feeding, transferring, toileting, continence, housekeeping, using a telephone, cooking, transportation, managing money, and the ability to administer medication as prescribed [23,24]. Some tasks require predominate cognitive skills such as managing money, self-administering medications, using the telephone, and others require sufficient motor skills such as walking and transferring [25]. The elements of functional status address basic activities necessary to live independently such as bathing, dressing, feeding, transferring, and toileting. Without the ability to conduct these functions, assistance is required to continue to live in the community. Each element of functional assessment has predictors for other limitations, different types of interventions and associated problems.

### 2.1. Transportation

Transportation can be a barrier to healthcare [26,27,28], a financial hardship [29], and a stressor for caregivers, especially when patients require regular medical attention [30]. Problems such as impairment in functional status, cognitive deterioration, and failing vision are associated with driving cessation [31]. The inability to drive or obtain transportation reduces the ability to live independently. Assessing specifically how patients travel to the grocery store and accomplish errands is important to healthcare management and is a vital aspect of the functional assessment.

### 2.2. Bathing and Dressing

Bathing and dressing are basic tasks that can be difficult, especially if a bathroom is not equipped with handles, raised toilet seats, and other adaptive devices that help reduce slip and fall injuries. Bathrooms tend to be dangerous areas of the house for older people [32] Bathtubs often require a high step over the side of the basin and can have wet, slippery surfaces which can enhance fall risk for people with reduced mobility, poor balance or neuropathies. The inability to bathe independently is a predictor of nursing home placement [33].

### 2.3. Preparing Meals

Physical and mental abilities are required to prepare meals. For physically compromised older patients diagnosed with COPD patients, 88% required assistance with cooking [34]. Among general community-dwelling elders, 13% report deficits with cooking [13]. Those with impaired cognitive ability and without caregiver support, using a stove can be dangerous. Using fresh ingredients or non-spoiled food can be difficult and reduced sense of smell or vision may restrict the ability to notice if food has spoiled. The ability to prepare meals can be a vital aspect of independent living.

### 2.4. Incontinence

Urinary incontinence is associated with a decline in general functional ability [35], falls [36], and is another prime factor in nursing home placement decisions [37]. A functional status assessment will help determine if incontinence exists, and possible causes. Incontinence is considered a geriatric syndrome, because of multiple confounders which make management difficult [38]. Approximately half of community-dwelling women aged 65 and over report urinary incontinence [39]. Fecal incontience should also be assessed. Fecal incontinence includes rectal leakage in the form of mucus and or stool.

### 2.5. Medication Administration at Home

Patients and caregivers frequently make mistakes when administering medications. The frequency of medication errors occurs up to 59% among community-dwelling elders [40]. The types of medication errors are associated with patient modification of the dosing orders (41.9%), faulty administration (31.8%), and not following clinical advice (21.7%) [41]. The majority of medication self-administration errors occur in people aged 80 years and over and those who have multiple diagnoses [42].

### 2.6. Money Management

Many seniors who are diagnosed with dementia do not relinquish managing the household finances even after mistakes have occurred [43]. In people aged 60 years and over, 11% have reported functional difficulty managing money and paying bills [13]. Even mild cognitive impairment can lead to problems managing money [44]. Discussions concerning the designation of another person to manage finances are delicate and difficult.

### 2.7. Using a Telephone

Using a telephone may not be a problem for many; however, poor vision, mental disability, and limited dexterity may reduce ability to operate a phone. Cell phones are largely replacing landlines in the United States and some of the large numbered telephones developed for people with poor vision and reduced dexterity are no longer available. According to the Pew Research Center, 23% of people aged 65 years and over do not use cell phones [45]. However, the younger old those who are highly educated and seniors of higher socioeconomic status tend to embrace technology. Most seniors over age 65 years do own and use a cell phone. Approximately, 77% of seniors reported being cell phone users in 2014, which is up from 69% in 2012 [45]. Understanding if a senior has the ability to call in case of emergency is an important element of the functional status assessment.

## 3. Benefits of Functional Assessment

Functional status assessment is helpful in understanding surgical risk and recovery [46] Older patients who have functional limitations preoperatively tend to have longer hospital stays along with unfavorable surgical outcomes [3], higher rate of mortality [47] and likely to experience readmission to acute care facilities [48]. Decisions regarding surgical *versus* medical management incorporate functional condition in addition to factors associated with general health and life expectancy [49]. Functional status scores along with other geriatric assessment measures can be used to determine surgical risk [50], one-year mortality [51], and changes in cancer treatment, such as intensification, decrease or delay in dose [52].

A functional status assessment provides the impetus for critical discussions among the family/support persons and the healthcare team about driving, living alone, managing money and health management options. Discussions concerning such issues create a great deal of tension among families. Loss of independence motivates transitions from living in a home of many years, to a smaller apartment or assisted living. For some families, nursing and physician clinical recommendations can help ameliorate anger or frustration for an older person who may be losing the ability to live independently.

It is important to remember that not every older person has a functional deficit. Appropriately, 23% of centenarians reported having no major chronic disease and 18% having no disability [53]. Many seniors exercise regularly, report high activity levels, and have no problems paying monthly bills [54]. Those aged 65–74 years (79%) had good or better health compared to those aged 85 years (67%) and over who reported good or better health [55].

## 4. Evaluating Functional Status

There are two ways to assess functional status which are self-report and performance evaluation. Many self-report instruments are valid and reliable and are used in clinical practice and research. Self-report instruments are tools equipped with questions the patient answers based on the ability to perform tasks. Performance evaluations are instruments used by nurses and physicians to empirically evaluate activities, such as gait, speed or grip strength.

### 4.1. Functional Assessment Cancer Therapy Scales

The Functional Assessment Cancer Therapy Scales (FACT) are instruments that measure quality of life with a functional assessment domain [56]). Many versions are developed for specific cancer diagnoses (the FACT-G is a general instrument) and the instruments are often used in research. The FACT is a reliable and valid 28-item self-assessment divided into four categories (physical, social/family, emotional, and functional well-being), and the internal consistency score is 0.89 [57]. The FACT-G has age related norms for scoring [58].

### 4.2. Short Form-36

Another quality of life instrument that also addresses a functional domain is the Short Form-36 [59]. Eight health-related concepts are included in the instrument: Physical functioning, social functioning, role limitation due to physical functioning, bodily pain, general health perceptions, vitality and role limitations caused by emotional problems, and mental health. The first four measures of physical health are combined to include a summary score of physical functioning. Generally, the SF-36 is a tool used in research and can help understand the relationship between physical functioning and emotional health. The instrument requires 15 min to complete. Scores are calculated and transformed to a 1 to 100 scale; with higher scores indicating better health. Reliability of the SF-36 scale has been assessed using Cronbach’s alpha coefficient with results ranging from 0.78 in general health perceptions to 0.91 in the physical functional domain. Discriminate validity has been demonstrated by the ability to classify subjects with and without other indications of poor health. The SF-36 is that is has age-related norms.

### 4.3. The Barthel Activity Index

The Barthel Activity Index is a scale commonly used in clinical practice to assess rehabilitation changes in stroke patients [60]. The Barthel consists of 10 items and the higher the score, the better the function. Bowel and bladder continence, feeding, grooming, dressing, transferring, toilet use, mobility, stairs, and bathing are the items included on the Barthel Activity Index and are intended to establish the level of independence. The internal consistency reliability for the Modified Barthel Index was 0.90, compared to 0.87 for the original scoring [61].

### 4.4. The Activity of Daily Living and Instrumental Activities of Daily Living

The Activity of Daily Living (ADL) scale is frequently used in clinical practice and part of a comprehensive geriatric assessment (CGA) used in inpatient and outpatient settings [23]. The ADL consists of six items and the instrument prompts the clinician to evaluate each task and determine whether a person is independent. If a person is dependent in any of the tasks, decisions concerning assistance and rehabilitation are then generally explored.

The Instrumental Activities of Daily Living (IADL) [24] addresses more refined activities as compared to the ADL scale. Using the telephone, getting to places beyond walking distance, grocery shopping, laundry, cleaning and housekeeping activities, and managing money are the six domains. The IADL has 11 items and is scored in that the patient earns three points for being able to perform a task without assistant, two points for some assistance, and one point for being completely unable. Dependence is defined by any one task that requires assistance. Validity was addressed using the Physical Self-Maintenance Scale (PSMS), Mental Status Questionnaire (MSQ) [62], Physical Classification (PC), Behavior and Adjustment rating Scale (BA) and all were significantly correlated at 0.1 with the exception of the BA scale. Reliability was established by two participants using the IADL scale on material from an interview (0.85) [24]. Current recommendations are to score IADL the same for men and women [63].

### 4.5. Fall Assessment

Falls are also related to functional status. The IADL tool is a predictor of falls in cancer patients [17] and the ADL tool is also predictive of falls in general geriatric populations [1]. The gold standard for fall screening is the simple question of whether a fall has occurred in the last six months [64]. If the patient has fallen within the last year a “Timed Get-up and Go” Test is performed [65].

## 5. Performance Evaluation

### 5.1. The Timed Get Up and Go Test (TGUAT)

The TGUAGT is performed by asking the person to rise from a straight backed chair and walk 10 feet, turn, return and sit down again. The patient is rated from a score of one (highest risk of fallings) to five (lowest risk of falling). The tests will be timed (under 10 s the patient is freely independent and over 30 s the patient is dependent on the assistance of others) [65]. The TUAGT [65] considers balance in the ability to rise from a sitting position and ambulate 10 feet and return to a sitting position. The TUAGT has been found to be correlated with falls [66].

### 5.2. Grip Strength

Grip strength is a performance measure that can help determine any possible limitations in mobility. Scores of three successive trials for each hand tested are recorded. The average score of the three trials can be compared to the normative data. From a statistical perspective, scores within two standard deviations of the mean are considered within normal limits. In addition, the individuals’ ability to use their hand functionally needs to be considered when interpreting a grip strength performance (Jamar handbook). Cutpoints are available from the manufacturer of the diameter and are specific to right or left hand and gender. Grip strength is associated with walking ability in hospitalized older patients [67]. Grip strength is also associated with poor physical performance in men [68].

### 5.3. Gait Speed

The six-minute walk test (6MWT) is reflective of functional ability [69] and can be considered a vital sign of general health [70]. The 6MWT is reflective of a person who requires total care to someone who is fit and less likely to be hospitalized [71]. Gait speed predicts hospitalizations [72] and mortality [73]. However, in chronic heart failure patients, the 6MWT is not clinical useful or predictive of cardiac function or exercise capacity [74]. Some parameters have been established for the test, use a walkway greater than five meters, asking the person to walk comfortably and use a stop watch for timing.

## 6. Management of Functional Status Limitations

The percentage of community-dwelling elders who recover from loss of function is estimated to be 81% [75]. Functional status limitations may be temporary and associated with a surgical procedure or as a result of an injury as compared to limitations associated with frailty, terminal illness or from chronic disease. People who are active and considered “fit” have more physical reserve and able to recover functional loss [76]. Recovering functional loss can require occupational/physical therapy, exercise, and regular movement and patients along with clinicians should be vigilant to encourage physical activity.

Determine if the limitations are mentally or physically inspired. Mild cognitive impairment can impinge on driving and handling money [44]. Depression can be assocaited with functional status changes. People who are diagnosed with depression are more likely to report problems with activities of daily living and cognitive changes [77]. The more symptoms of depression, the more functional limitations experienced by the patient [78]. Managing depression will help improve functional abilities [79].

Many older patients experience functional decline during hositalization. In a Taiwanese study, up to 74% of older people develop functional impairment during a hospital admission and 32% of those had an impairment six months after discharge [80]. Bedrest is a prime feature in transitioning from no disability to severe disability [81]. It is important to maintain sensory stimulation and regular walking regiemes [82]. Nurses and the entire healthcare team must play a role in the maintenance of functional status. Recognizing that a hospitalization can be detrimental to the physical ability of an older person, hospitals must embrace education and promotion of physical function [83]. Offering function-focused care can help perserve functional status while in the hospital [84]. All healthcare team members should be vigulant reguarding medications and potential drug errors which can have an effective on functional status [85].

Trends in functional status scores can help the clinical team make treament decisions. Functional status assessments should be conducted regularly to understand the burden of disability [75]. A single functional assessment can be helpful, but does not provide the comprehensive understanding of capability. Treatments and progression of disease are reflected in functional status scores and can help with decisions about dosing, imaging studies and other elements of medical management.

For problems associated with postsurgical procedures or an injury, physical therapy can be a reasonable strategy. Working to not lose any function and regain what was lost is important to recovering independence. Routine physical therapy can be helpful in addressing muscle weakness and flexibility; however patients must maintain the exercises regularly even after therapy sessions have completed [86]. Adherence among older adults to exercises recommended by the physical therapists unfortunately tends to be poor [87]. Despite the benefits of physical therapy, only few ever access occupational/physical therapy despite Medicare coverage [88]. Barriers to outpatient physical therapy include poor social support, depression, low activity lifestyle, anxiety, and low self-efficacy [89].

Organized exercise and movement programs are also beneficial in enhancing functional [86]. Tai Chi is effective, when engaged regularly for eight to 12 months [90] in improving strength and endurance [91], as well as balance and flexibility [92]. Yoga is beneficial [93] and many continue to adhere to regular participation [94]. General aerobic exercise is effective in enhancing ambulatory status and gait [95,96,97].

To reduce the potential for functional status limitations after surgery, adequate nutrition is important for recovery [98]. Body Mass Index (BMI) influences muscle strength, as well as physical function in older people [99] Malnutrition is common in older people and is reflective of poor performance status. Older people with higher BMI tend to have more functional status impairment [100]. Addressing nutrition can help improve functional and physical outcomes [101].

## 7. Conclusions

Functional status assessment and management are critical to the care of the older person. Incorporating functional assessment in ambulatory care facilities can help medical management strategies, coping with disability and patient/family interactions. Many clinical instruments exist that are either self-report or performance based that require brief amounts of time to conduct. Interventions range from treatment of the underlying cause, to physical therapy or participation in regular exercise. Preservation of independence is a critical feature of care for the older person and functional status assessment is part of that effort.
